# Grapevine rootstock and soil microbiome interactions: Keys for a resilient viticulture

**DOI:** 10.1093/hr/uhac019

**Published:** 2022-02-19

**Authors:** Romain Darriaut, Vincent Lailheugue, Isabelle Masneuf-Pomarède, Elisa Marguerit, Guilherme Martins, Stéphane Compant, Patricia Ballestra, Steven Upton, Nathalie Ollat, Virginie Lauvergeat

**Affiliations:** EGFV, Univ. Bordeaux, Bordeaux Sciences Agro, INRAE, ISVV, F-33882, Villenave d'Ornon, France; EGFV, Univ. Bordeaux, Bordeaux Sciences Agro, INRAE, ISVV, F-33882, Villenave d'Ornon, France; Université de Bordeaux, UMR Oenologie 1366, INRAE, Bordeaux INP, Bordeaux Sciences Agro, ISVV, Villenave d'Ornon, France; Bordeaux Sciences Agro, 33170 Gradignan, France; EGFV, Univ. Bordeaux, Bordeaux Sciences Agro, INRAE, ISVV, F-33882, Villenave d'Ornon, France; Université de Bordeaux, UMR Oenologie 1366, INRAE, Bordeaux INP, Bordeaux Sciences Agro, ISVV, Villenave d'Ornon, France; Bordeaux Sciences Agro, 33170 Gradignan, France; AIT Austrian Institute of Technology, Center for Health and Bioresources, Bioresources Unit, Konrad Lorenz Straße 24, Tulln, A-3430, Austria; Université de Bordeaux, UMR Oenologie 1366, INRAE, Bordeaux INP, Bordeaux Sciences Agro, ISVV, Villenave d'Ornon, France; Bordeaux Sciences Agro, 33170 Gradignan, France; EGFV, Univ. Bordeaux, Bordeaux Sciences Agro, INRAE, ISVV, F-33882, Villenave d'Ornon, France; EGFV, Univ. Bordeaux, Bordeaux Sciences Agro, INRAE, ISVV, F-33882, Villenave d'Ornon, France

**Keywords:** vine health, terroir, sustainable viticulture, soil diversity, rhizosphere, plant growth-promoting rhizobacteria, microorganisms’ interactions, microbiome engineering, grapevine rootstock, Environmental stress

## Abstract

Soil microbiota has increasingly been shown to play an integral role in viticulture resilience. The emergence of new metagenomic and culturomic technologies has led to significant advances in the study of microbial biodiversity. In the agricultural sector, soil and plant microbiomes have been found to significantly improve resistance to environmental stressors and diseases, as well as influencing crop yields and fruit quality thus improving sustainability under shifting environments. Grapevines are usually cultivated as a scion grafted on rootstocks, which are selected according to pedoclimatic conditions and cultural practices, known as terroir. The rootstock connects the surrounding soil to the vine’s aerial part and impacts scion growth and berry quality. Understanding rootstock and soil microbiome dynamics is a relevant and important field of study, which may be critical to improve viticulture sustainability and resilience. This review aims to highlight the relationship between grapevine roots and telluric microbiota diversity and activity. In addition, this review explores the concept of core microbiome regarding potential applications of soil microbiome engineering with the goal of enhancing grapevine adaptation to biotic and abiotic stress.

## Introduction

Omics technologies have deepened our knowledge and understanding of telluric and ecosystemic processes; these developments underscore the importance of soil microbiome to plant health. The microbiome has recently been redefined as the microbiota and its theater of activity which combine microbial structural elements such as proteins, peptides, lipids, nucleic acids, polysaccharides, and microbial metabolites as signaling molecules, toxins, (in)organic molecules, and the environmental conditions [1]. Currently, the primary methods used to explore the taxonomic and functional soil microbiome diversity utilize plating methods and computed metagenomics which respectively rely on media composition and high-throughput sequencing [2]. Through the use of these techniques, it has been suggested that plant-associated microorganisms are recruited from the soil microbiota, thus serving as the microorganisms’ reservoir of rich microbial diversity [3].

In viticulture, the soil microbiome is now considered as a terroir component that could influence grape berry composition [4]. Studying the microbiome in vineyards, especially fungi and bacteria, is an emerging field of science as it holds the potential to improve grapevine adaptation to climate change and prevention of pathogenic infection. Thus, the study of vineyard microorganisms holds tremendous potential for improving vine resilience and helping vineyards better face increasing environmental stress.

The composition of the soil microbiota, and therefore its related biological activity, is dependent on many factors (*e.g.* physicochemical characteristics of the soil, plant species and cultivars, climatic conditions, cultural practices …) [5, 6]. Regardless of the microbiota already present in the soil, the main drivers of the composition of the microbial community associated with the root system (epiphytic and endophytic) are the primary and secondary metabolites exudated by the roots [7]. The composition of the exudates vary depending on environmental factors, as well as plant species and cultivars [8, 9], which collectively shape the root microbiome.

Cultivated grapevines are typically grafted plants composed of a scion (*Vitis vinifera* L.), which produces grape berries, and a rootstock (*Vitis* spp., tolerant to phylloxera aphids), which is selected considering pedoclimatic conditions. Grafting is a practice widely used to improve resistance to environmental stresses, yield and quality of the harvested product [10]. The rootstock works as an interface between the soil and the grapevine-associated microbiota, hence modulating the plant holobiont. The scion cultivar is another factor in this complex rootstock x scion × soil interaction, which may influence the root-associated microbiome. The rootstock’s capacity to interact with soil microorganisms differs between genotypes due to their intense breeding and genetic background histories [11]. Rootstocks display contrasting root system in terms of root architecture, as well as synthesis and exudation of metabolites. Some of these compounds are signaling molecules, which shape and attract soil microorganisms. It is therefore essential to understand the role of the rootstock in these interactions that could be further utilized to isolate and promote biofertilizers and bioprotectors. Moreover, the use of rootstocks appears to be an appropriate strategy to conserve wine quality produced by the scion while simultaneously conferring resistance to biotic and abiotic constraints [12]. This review serves to update and expand upon the role of soil microbiome and rootstock dynamics in improving grapevine resilience.

## Close to the roots, a dynamic spot for molecular exchange

### The soil acts as a microbial reservoir for the plant

The grapevine microbiome has been investigated in every compartment using culture-dependent and independent techniques. Independent of soil type and cultivar genotype, the prokaryotic microbiome of *V. vinifera* is mainly composed of *Proteobacteria*, followed by *Firmicutes*, *Actinobacteria*, *Acidobacteria*, and *Bacteroidetes* (Table S1). The grapevine’s eukaryotic microbiome consists of *Ascomycota* and *Basidiomycota* on both the above and below-ground parts of the vine (Table S2) while the *Glomeromycota* division is established in the vine roots. Wei et al. (2018) [13] found in their multi-compartment study that *Proteobacteria* and *Firmicutes* are more common to berries, leaves, and grape must, whereas *Bacteroidetes* and *Actinobacteria* adapt better to soil. The authors found that even in the phyllosphere, which is the target of several air-borne pathogens, the relative abundance of bacterial genus and class depends on the plant organs.

**Figure 1:**
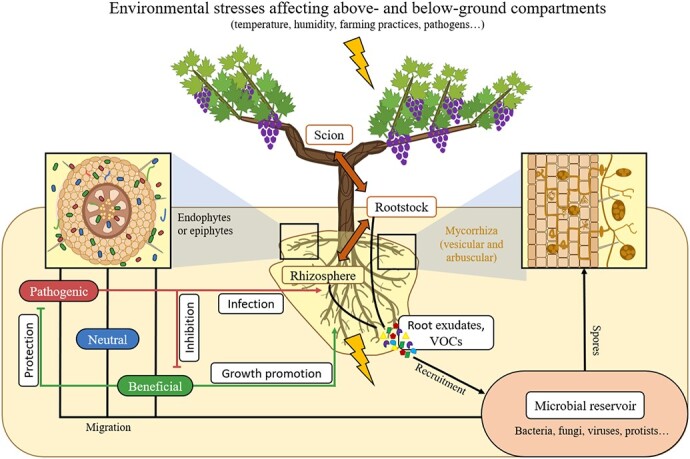
Schematic representation of the vine-soil interactions. Environmental stresses afflict both below and above ground compartments of vine. Scion and rootstock communicate through long distance signaling compounds. These signaling pathways modulate the root exudates composition (*e.g.* VOCs, Volatile Organic Compounds) into the soil microbial reservoir. Microorganisms are therefore chemoattracted and present pathogenic, neutral or beneficial functions towards the vine. They can be either epiphytic and/or endophytic (box on the left), such as mycorrhizal fungi (box on the right).

The rhizosphere, defined as the tight area of soil enveloping the plant roots, hosts a tremendous number of microorganisms, which interact directly or indirectly with the plant. This soil compartment supports a complex microbiome and is considered as one of the most dynamic ecosystems on Earth. Part of the rhizosphere microbiome, also known as rhizomicrobiome, has been shown to provide the host plant with better capacities to adapt to environmental stresses, potentially playing an integral role in plant health [14]. Soil microflora is mainly composed of bacteria, archaea, fungi, protists, and viruses, which have either beneficial, neutral, or pathogenic relationships with the plant (Fig 1). Pathogenic microorganisms participate in the root infection processes whereas beneficial microbiota promote the plant’s growth and defense mechanisms [5].

The relative abundance of bacterial and fungal rhizomicrobiome varies with scion/rootstock combination features, soil type, climatic conditions, soil depth, and cultural practices [15–19]. Among fungi, the most encountered taxa in the vineyard soil are principally from the *Ascomycota* and *Basidiomycota* phyla (Table 1). With regard to bacteria, the most abundant genera found in the grapevine rhizosphere belong to *Proteobacteria*, *Actinobacteria*, *Firmicutes*, *Bacteroidetes*, and *Acidobacteria* phyla.

**Table 1:** Examples of the main bacterial and fungal taxa found in the rhizomicrobiome of grafted and ungrafted grapevine, with their relative abundances and associated sequencing target region

**Major bacterial taxa (% of relative abundance), and the associated target region**	**Major fungal taxa (% of relative abundance), and the associated target region**	**Studied scion/rootstock combination**	**Reference**
	Root / surrounding soil (ITS1): *Ascomycota*, *Mortierellomycota*, *Basidiomycota*. Relative abundances not provided	Pinot noir cv. (*Vitis vinifera*). Presence or absence of rootstock not provided.	(Liu *et al*., 2021) [20]
Rhizosphere (16S V4-V5): *Acidobacteriota* (35%), *Proteobacteria* (22%), *Latescibacteriota* (15%), *Methylomirabilota* (6%), *Gemmatimonadota* (4%)		Ungrafted 1103P, 140 Ru, 161–49 C, and Kober 5BB cv.	(Dries *et al*., 2021) [21]
Rhizosphere (16S V3-V4): *Proteobacteria* (~45%), *Bacteroidetes* (~15%), *Firmicutes* (~9%), *Actinobacteria* (~7%), *Acidobacteria* (~6%)	Rhizosphere (ITS1): *Ascomycota* (~47%), *Basidiomycota* (~15%), *Mortierellomycota* (~10%)	Ungrafted Malbec (*V. vinifera*) and Cabernet Sauvignon cv.	(Aguilar *et al*., 2020) [22]
Rhizosphere (16S V4): *Proteobacteria* (~70%), *Actinobacteria* (~18%), *Bacteroidetes* (~8%), *Firmicutes* (~5%)	Rhizosphere (ITS1): *Ascomycota* (~50%), *Basidiomycota* (~45%)	Syrah cv. (*V. vinifera*) grafted on 1103P	(Deyett & Rolshausen, 2020) [23]
Rhizosphere (16S V4): *Proteobacteria* (27%), *Actinobacteria* (21%), *Acidobacteria* (15%), *Bacteroidetes* (6%)	Rhizosphere (ITS2): *Ascomycota* (67%), *Basidiomycota* (16%), *Zygomycota* (12%)	Tempranillo (*V. vinifera*) cv. grafted on 110R, 140 Ru, 1103P (all above are *Vitis berlandieri* × *V. rupestris*), 41 B (*V. vinifera* × *V. berlandieri*), and 161–49 C (*V. riparia* × *V. berlandieri*)	(Berlanas *et al*., 2019) [24]
	Rhizosphere (ITS2): *Ascomycota* (61%), *Basidiomycota* (21%)	Tempranillo cv. grafted on 110R	(Martínez-Diz *et al*., 2019) [25]
Root and Rhizosphere (16S V3- V4): *Proteobacteria* (53%), *Actinobacteria* (24%), *Bacteroidetes* (5%), *Chloroflexi* (4%), *Acidobacteria* (4%)		Barbera cv., ungrafted (*V. vinifera*) and grafted on SO4, 420A, 161-49C and 157-11C (all are *Vitis riparia* × *V. berlandieri*)	(Marasco *et al*., 2018) [15]
Rhizosphere (16S V1-V4): Actinobacteria (52%), Proteobacteria (36%), Gemmatimonadetes (2%), *Bacteroidetes* (~2%)		Pinot noir cv. Presence or absence of rootstock not provided.	(Novello *et al*., 2017) [26]
Rhizosphere (16S V5-V7): *Actinobacteria* (47%), *Proteobacteria* (22%), *Bacteroidetes* (13%)		Zweigelt cv. clone GU4 (*V. vinifera*) grafted on Kober 5BB (*V. berlandieri* x *Vitis riparia*)	(Samad *et al*., 2017) [17]

**Table 2:** Non-exhaustive list of common biological control products used in the wine-growing industry to apply on the grapevine’s foliar part

**Microorganism as active ingredient**	**Target pathogen**	**Tradename (manufacturer)**	**Mode of action**	**Reference**
*Bacillus subtilis*	*Botrytis cinerea*	Rhapsody® Serenade Max® (Bayer)	Antimicrobial, eliciting plant defense	(Thomidis *et al*., 2016) [89]
*Bacillus pumilus*	*Uncinula necator*	Sonata ® (Bayer)	Antimicrobial, antibiosis	(Serrano *et al*., 2013) [90]
*Streptomyces griseoviridis*	*Botrytis cinerea*, *Fusarium*, *Alternaria*	Mycostop ® (Verdera)	Competition	(Lahdenperä *et al*., 1991) [91]
*Ampelomyces quisqualis*	*Uncinula necator*	AQ10 ® (Ecogen)	Competition, antibiosis	(Hofstein *et al*., 1996) [92]
*Trichoderma harzianum*	*Botrytis cinerea*	Trichodex ® (Makhteshim-Agan)	Competition	(O’Neill *et al*., 1996) [93]
*T. atroviride*	*Phaeoacremonium minimum*, *Phaeomoniella chlamydospora*, *Botrytis cinerea*	Vintec ® (Belchim Crop Protection)	Antibiosis	(Pertot *et al*., 2017) [100], (Pertot *et al*., 2016) [111]
*Saccharomyces cerevisiae*	*Botrytis cinerea*	Julietta® (Agrauxine)	Antibiosis	(São-José *et al*., 2017) [94]
*Metschnikowia fructicola*	*Botrytis cinerea*	Noli ® (Koppert Biological Systems)	Antimicrobial, eliciting plant defense	(Sipiczki *et al*., 2006) [95]
*Aureobasidium pullulans*	*Botrytis cinerea*	Botector® (Nufarm)	Competition	(Calvo-Garrido *et al*., 2019) [96]

These phyla are keystone taxa that perform a broad range of functions in the soil ecosystem [27]. Zarraonaindia *et al.* (2015) [18] and Marasco *et al.* (2018) [15] showed an enrichment of the rhizosphere compared to bulk soil for main phyla such as *Gammaproteobacteria*, *Betaproteobacteria*, and *Actinobacteria*. This increase in bacterial richness might be promoted, through the use of flagella, by chemoattractants (*e.g.* sugars, amino acids, organic acids, vitamins, phytohormones, flavonoids, terpenes) [28]. Indeed, genes involved in bacterial chemotaxis and motility as well as flagella association, are more present in microbial communities found in root-associated environments, in comparison to bulk soil [29]. Root microbial communities in grapevines were also investigated using 16S/ITS rRNA amplicon sequencing, shotgun metagenomics, and cultivable approaches [30]. It appears that bacterial diversity is lower in the root compartment than in the rhizosphere, and the majority of root-associated bacterial taxa matched the bacteria found in the soil [15, 18], which also occurs with fungal diversity [25, 31], highlighting soil microbial reservoir capacity.

### Soil and rhizosphere: A microbial source of inoculum of grape berry microbiota

Must and wine microorganisms belong mainly to the microbial consortia of grape berries [32]. Many studies support that the main source of these microorganisms is the vineyard soil [18, 33], even though the atmospheric microbiome also influences the composition of fungal and bacteria communities associated with leaves, flowers, and fruits [34]. The root endophytes can shape the microbial community of aboveground organs by changing endophytic microbial loads in grapes [18]. A significant input of soil microorganisms to grapes through epiphytic migration during harvest was also suggested [35]. Contrary to the bacterial component, studies on vineyard soil contribution to the yeast community of grapes are scarce. A hypothetical endophytic way of colonization was proposed for the fermentative yeast *Saccharomyces cerevisiae* to be transported from the soil via roots and stems to the surface of the grape berry [36] as shown for bacteria [37]. As for bacteria, vineyard soil appears to be a permanent natural reservoir of non-*Saccharomyces* yeasts via possible contamination of grapes with edaphic microorganisms due to deposit of dust from vineyard soil [32]. Microbial communities on grapes could have the potential to influence grape composition and thus the organoleptic properties of the wine, contributing to a regional terroir. Zarraonaindia *et al.* (2015) [18] showed that the aboveground bacterial community was significantly influenced by soil edaphic factors such as total carbon, moisture, and soil temperature, which would ultimately impact the quality of grapes due to changes in nutrient availability for the plant. Weather and soil properties influence soil and must microbial diversity that will indirectly impact wine aroma profiles [38]. The contribution of the soil microbial component on the berry and the final wine composition should be evaluated in light of other factors including pedoclimatic, human parameters, rootstock and scion genotypes that define the concept of terroir.

### The impact of telluric microbiota on grape berry composition

In agriculture, plant probiotic bacteria significantly impact crop quality and fruit composition by increasing vitamins, flavonoids, and antioxidants content, among other benefits [39]. For example, the addition of a Plant-Growth Promoting Bacterium (PGPB) *K. radicincitans* modifies amino acid, sugar, and volatile composition of ripened tomato fruits, thus contributing to a more pleasant-tasting fruit [40]. Aoki *et al.* (2017) [41] investigated the activation in grape berries of the gene expression of stilbene synthase, a key enzyme in resveratrol synthesis, by a *Bacillus cereus* strain. Native microorganisms can exert an accumulation of volatile compounds in grape berries that could be activated by phytopathogens in the case of volatile precursors of volatile thiols (3MH) responsible for grapefruit aroma in white wines [42]. The production of aroma by grape-associated microorganisms could also directly impact grape berry composition [43].

Grape berry endophytic and epiphytic microorganisms are known to activate metabolic pathways leading to an increase in phenolic compounds or other aroma compounds biosynthesis, as reviewed in Otoguro and Suzuki (2018) [42]. Even if the endophytic berry microbial community is largely derived from the soil, very few studies evaluate the impact of telluric microbiota on berry composition and are mainly focused on arbuscular mycorrhizal fungi (AMF).

By using Biolog™ EcoPlates technology, Ji *et al.* (2019) [44] showed a correlation between metabolic activities and functional diversity of rhizosphere microbial communities and physicochemical indices of grape berry quality. Association of grapevine with AMF facilitates the synthesis of plant secondary metabolites such as resveratrol, flavonol or anthocyanin, which improve berry quality and plant tolerance to environmental stresses [45]. Wine produced from a vineyard with cv. Sangiovese had better oxidative stability and a significantly higher level of bioactive compounds such as gallic acid, resveratrol, caffeic acid and, quercetin, when treated with a consortium of *Glomus* species plus soil bacteria, fungi and, yeast to a lesser extent, compared to the wine produced by control vines [46]. The protective role of AMF against warming effects on berries on three clones of Tempranillo was shown to improve their antioxidant properties and anthocyanin content [47]. The inoculation of eight ancient grapevine varieties with a mixture of five AMF species reduced the berry mass and increased the soluble sugars and anthocyanin contents for most of the cultivars [48]. The intensity of these variations on berries was different among the cultivars, suggesting a genotype dependent effect. These studies do not take into account the effect of the rootstock genotype as almost all were performed with ungrafted cultivars. Therefore, the functional potential of the rootstocks to impact the soil microbiota effect on fruit physiology, susceptibility to pathogen and grape berry quality remains to be explored.

### Root-associated and rhizosphere microbiomes are regulated by grapevine genotype and possess useful plant growth-promoting features

Plant species and genotypes play determinant roles in selecting the telluric microorganisms that will surround the host. As most cultivated grapevines are chimeric plants composed by *V. vinifera* cultivars grafted on American *Vitis* species and hybrids, it is essential to consider the effect of the scion/rootstock combination. To date, only one study analyzed the bacterial community structure in the rhizosphere of 4 cultivars × 4 rootstocks combinations [49]. Authors showed that the diversity of rhizosphere bacteria is impacted first by the cultivar followed by rootstock genotypes, but the effect was dependent on the diversity index used. The distinct genetic component and capacity to produce photosynthate components of the cultivars might alter the exudate composition and could explain this difference in bacterial diversity. Bacterial microbiomes in the rhizosphere of five different rootstocks grafted with the same Barbera cv. were significantly different in terms of richness, diversity, and community networking, within the same vineyard [15]. Biget *et al*. (2021) [50] demonstrated through their multi-site analysis within a vineyard that vine age was one of the main drivers of bacterial and fungal root endophytes, even though the genetic background of rootstock was not investigated. Considering this, Berlanas *et al.* (2019) [24] highlighted that rootstock genotype had a greater impact than millesimal or sampling date on bacterial and fungal microbiome structure in the rhizosphere exclusively in mature vineyards. Predominant amounts of *Proteobacteria* and *Actinobacteria* were found in all samples of rhizosphere, but bacterial genera varied depending on the rootstocks. With regard to fungi, the *Ascomycota* and *Basidiomycota* phyla varied greatly among rootstocks. Specific genera were affiliated to distinct rootstock genotypes, such as *Geopyxis* for the 110R rootstock, or *Clonostachys* for 1103P and 140 Ru rootstocks.

Regarding functional screening of indigenous isolates, Samad *et al.* (2017) [17] and Marasco *et al.* (2018) [15] confirmed the significant enrichment of *Proteobacteria* in grapevine root tissues (Kober 5BB rootstock, and ungrafted/grafted Barbera cv. on 402A, 157-11C, SO4, 161-49C, respectively), while *Actinobacteria* and *Bacteroidetes* remained at relatively constant levels in both rhizosphere and root compartments. Conversely, *Gemmatimonadetes* and *Firmicutes* were less abundant in roots than the surrounding soils. In both studies, Plant-Growth Promoting (PGP) activities of strains belonging to the *Enterobacteriaceae* and *Pseudomonadaceae* families were tested for production of hydrogen cyanide, ACC deaminase (ACCd), siderophores, indole acetic acid (IAA), and for phosphate solubilization. It has been shown by Marasco *et al.* (2018) [15] that PGP functional genes were conserved in both the rhizosphere and root endosphere despite selecting different bacterial communities, and therefore that the frequencies of these PGP traits were not dependent on the rootstock genotype. For Syrah cv. grafted on 1103P rootstock, Deyett and Rolshausen (2020) [23] observed a different enrichment composed mainly of *Rhizobium*, *Devosia*, *Streptomyces*, and *Pseudomonas* genera in the rhizosphere. This study also revealed that fungal and bacterial richness in roots accounted for 64% of the amplicon sequence variants (ASVs) found in the rhizosphere and soil compartments. *Streptomyces* and *Pseudomonas* genera are often associated with PGP activities but also inhibit the colonization of pathogens in grapevine woods [51]. Using a disruptive approach based on metaproteomic, Bona *et al.* (2019) [52] confirmed that the high biochemical activity (*i.e.* phosphorus metabolic processes and regulation of nitrogen compounds) in the rhizosphere of ungrafted *V. vinifera* cv. Pinot noir was largely attributed to bacteria belonging to the *Proteobacteria* phylum. To another extent, D’Amico *et al.*, (2018) [53], observed a depletion and sometimes a total absence of potassium (K) solubilizing bacterial members from the *Micrococcaceae*, *Comamonadaceae*, *Cytophagacea*, *Sphingomonadaceae*, *Rhizobiaceae*, *Xanthomonadaceae*, and *Microbacteriaceae* in the rhizosphere and roots of 1103P rootstock, whereas they were detected in 5BB rootstock with the same Lambrusco cultivar. This dysregulation of the functional microbiome was linked to the problem of K absorption observed in the studied *Vitis berlandieri* × *Vitis rupestris* rootstocks. Except for AMF, more studies have been focused on the bacterial communities of grapevine roots and rhizosphere compared to studies of fungal communities. Given the importance of rhizosphere functions, it is relevant and crucial to examine the link between rootstock agronomic features and rhizosphere microbiome traits.

### Case of the famous symbiont, the arbuscular mycorrhizal fungi

AMF symbioses are endomycorrhizal associations with obligate biotrophic fungi belonging to the *Glomeromycota* division. This is the most frequently encountered mycorrhizal form encompassing grapevines as approximately 80% of terrestrial plants are able to associate with AMF [37, 38, 39]. AMF symbioses are mainly induced in soil where P availability is low, and play a key role in providing P and N to plant root cells, which can be attributed to increased soil exploration surface due to extra-radicular hyphae proliferation [55]. In return, fungi receive photosynthetically fixed carbon assimilated from plant cells. AMF do not only affect plant growth traits, water and nutrient uptake, but also protect their host from pathogens. Since the first description of two AMF species by Tulasne et Tulasne in 1845, more than 260 Glomeromycotan species have been discovered [56]. The most common species identified using culture-dependent approaches are included in the *Glomeraceae* order such as *G. intraradices* or *G. mosseae*. New technologies based on molecular approaches provided deeper insights about AMF diversity in vineyards by sequencing ribosomal Internal Transcribed Spacers (ITS) or their small subunit (SSU) rRNA fragments [57, 58]. Drain *et al.* (2019) [59] proposed a standardized protocol to study AMF communities from root samples of vines. The authors amplified the D2 domain from the Large Subunit Region (LSU) and revealed the predominance of the *Rhizophagus* and *Glomus* genera coupled to eight other genera from the *Glomeromycota* division. However, a clear picture of how AMF diversity colonizes grapevine roots in different parts of the world is incomplete, especially since the classification of AMFs remains controversial and molecular techniques for their identification have not been standardized [60].

Although it is assumed that sustainable practices enhance the spore abundance and diversity of AMF [61], they are influenced by several factors including edaphic parameters and grapevine genotype. Moukarzel *et al.* (2021) [62] demonstrated a significant difference in the AMF community associated with nine rootstocks grafted or not with Pinot noir cv. using denaturing gradient gel electrophoresis (DGGE) and trap cultures. Nerva et al. (2021) identified the influence of the rootstock genotype in activating distinct defense pathways by young cuttings, grafted on either 1103P or SO4 rootstock, when treated with *Rhizophagus irregularis* and *F. mosseae* [63]. While studies of citrus have shown scions to be more influential to the AMF community structure than on rootstock [64], the role that scion genotype could play in AMF diversity in grapevines has yet to be explored. The selection of rootstock and scion genotype are important in determining grapevine capacity to form mycorrhizal associations that could enhance host mineral uptake and increase grapevine sustainability.

## Microbiome engineering, a tool to promote plant health

### The concept of compositional and functional core microbiome

The concept of core microbiome relies on operational taxonomic units (OTUs), and to some extent on ASVs, shared between different individuals of the same species, as was first proposed in humans [65]. Despite its complexity, the concept of core microbiome is gaining support and several definitions have been made with regard to either microbiome’s functionality, temporal stability, taxonomy, plant-adapted, or ecology [66]. Most of the time, core microbiome is referred to as the compositional core based on taxonomy or functional core. Indeed, this core concept is not only considered as the microorganism’s diversity, but also as the core interactions that are used to maintain an individuals’ health, and on a larger scale the ecosystem. Crops and plants in general, are associated with distinct soil microbiomes which are influenced, independent of temporal factors, by biotic and abiotic components [67].

**Figure 2:**
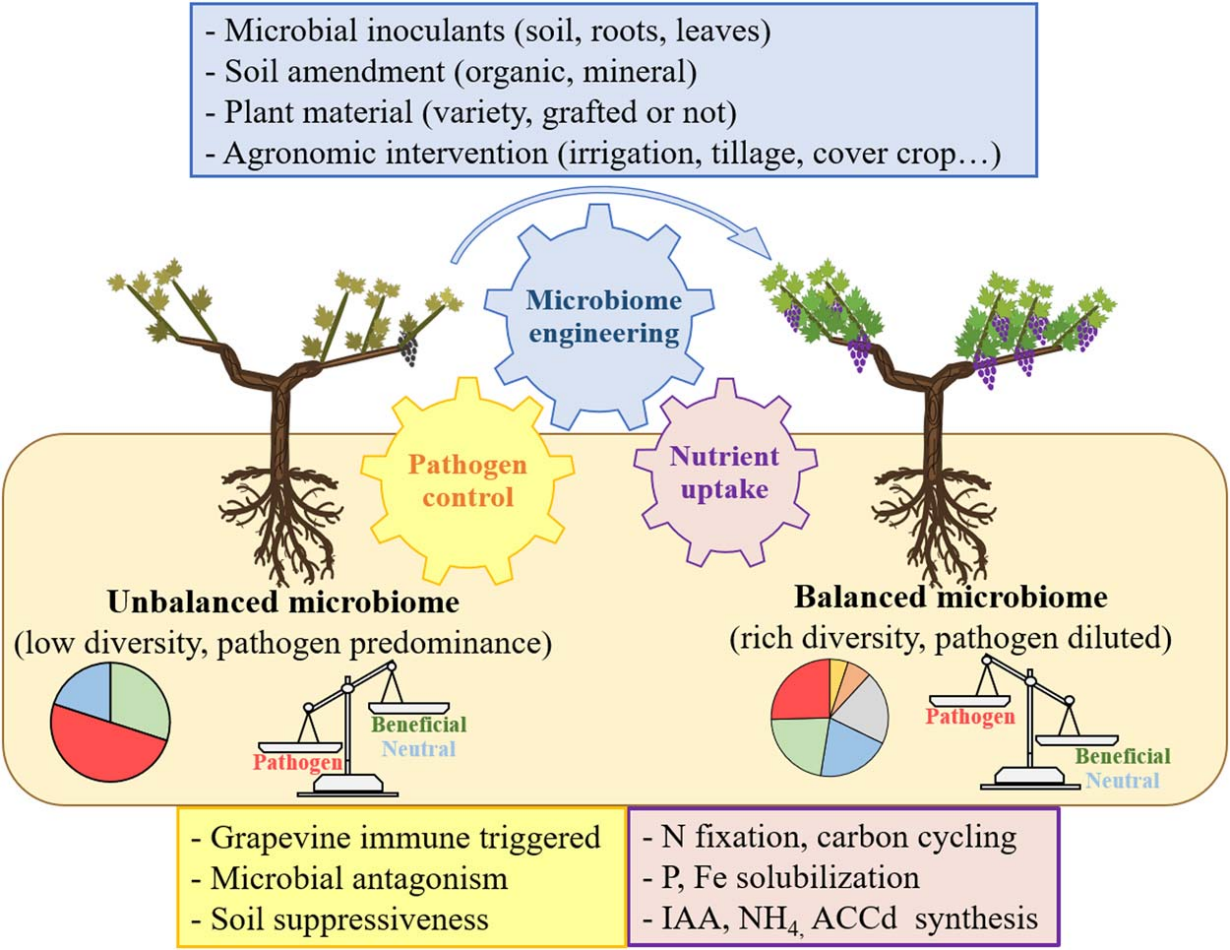
Schematic representation of grapevine health affected by soil microbiome services, pathogen control (yellow box) and nutrient uptake (purple box), which are enhanced by microbiome engineering (blue box). Unbalanced microbiome comes along with a low microbial diversity with predisposition to pathogen predominance, while high microbial diversity is found in balanced microbiome and inhibits the pathogen capacity to afflict grapevine.

Swift *et al*. (2021) [68] suggested, subsequently to a multi-compartment analysis submitted to irrigation stress, that the core microbiome is quite conserved in the different analyzed rootstocks (cv. Chambourcin grafted on 1103P, 3309C, and SO4). The different irrigations lead to microbial changes in aerial compartments such as different amounts of *Acetobacterales* and *Saccharomycetes* in berries which could affect wine quality. Carbone *et al*. (2021) [69] recently pointed out this shift in fungal communities under three distinct irrigation regimes (25%, 50%, or 100% of field capacity) with 22.3% of fungal OTUs shared in roots among those conditions, while 66.8% and 55.6% OTUs were found to be common in rhizosphere, and bulk soil compartments, respectively. Despite neglecting the role of rootstock, Liu & Howell (2021) [20] unveiled the fungal core microbiome in Merlot cv. which displays 32.75% of shared OTUs between roots and soil, fluctuating in abundance across the season. This supports the idea that the grapevine core microbiome relies on the composition of microbial soil reservoir, which is recruited differently according to the rootstock.

Core functions such as biogeochemical processes in the soil appear to be related to taxonomically distinct patterns but with similar metabolic functions, hence confirming that the theater of microbiota activity can be distinguished into taxonomy and functioning that interact with the terroir [38]. Terroir is a broad concept that can be described as the components driving the aromas and wine typicity within a defined geographical region with specific soil topology, and viticultural practices including cultivar variety [70]. As discussed previously, different rootstocks are able to be associated with different microbial communities sharing similar functional traits [15, 53]. Functional redundancy is indeed the idea that more than one taxon can exert the same function within a microbial community [71]. Unravelling the core species recruited through rootstocks could be a powerful tool in determining microbiome responses to environmental constraints. Therefore, microbiome functioning must be understood in order to predict plant health in response to various stresses, even though microbiome-plant partnerships are complex belowground-based interactions linked with the soil.

**Table 3:** List of inocula used for their biological control properties on grapevine and applied on the soil or root system

**Target pathogen (Disease)**	**Inoculum identification (Origin)**	**Observations**	**Plant material** **(Type of application)**	**Reference**
*Botrytis cinerea* (Gray mold)	*Bacillus subtilis* PTA-271, (Grapevine rhizosphere) *Pseudomonas fluorescens* PTA-CT2, and (Grapevine stem) *Pantoea agglomerans* PTA-AF2 (Grapevine leaf)	Systemic resistance. Accumulation of stilbenic phytoalexins, *trans*-resveratrol and ε-viniferin in leaves and berries.	Field, 15 years-old cv. Chardonnay-41B (Soil drenching)	(Aziz *et al*., 2016) [107]
	*P. agglomerans* Pa-AF2, (Grapevine leaf) *Acinetobacter lwoffii* Al-113, (Grapevine roots) *Bacillus subtilis* Bs271, and (Grapevine rhizosphere) *P. fluorescens* PfCT2 (Grapevine stem)	Local and systemic resistance. Early oxidative burst and stilbenic phytoalexins (*trans*-resveratrol and *trans*-ε-viniferin) accumulation in leaves.	*In vitro*, 4 weeks-old cv. Chardonnay (Root dipping)	(Verhagen *et al*., 2011) [104]
	*B. subtilis* PTA-271, *A. lwoffii* PTA-113, *P. agglomerans PTA-AF1* and *PTA-AF2*, and *P. fluorescens* PTA-268 and PTA-CT2 (All isolated from grapevine rhizosphere)	Systemic resistance. Accumulation of chitinase and β-1,3-glucanase in leaves and berries.	Field, 10 years-old cv. Chardonnay-41B (Soil drenching)	(Magnin-Robert *et al*., 2007) [105]
	*Burkholderia* sp. BE17 and BE24	Systemic resistance. H_2_O_2_ accumulation and upregulations of *PR5* and *PR10* in leaves.	*In vitro*, 4 weeks-old cv. Chardonnay (Root dipping)	(Esmaeel *et al*., 2020) [106]
	*Paraburkholderia phytofirmans* PsJN	Systemic resistance. H_2_O_2_ accumulation and upregulations of *PR1*, *PR2*, *PR5*, *WRKY*, and *JAZ* in leaves.	*In vitro*, 4 weeks-old cv. Chardonnay (Root dipping)	(Miotto-Vilanova *et al*., 2016) [107]
*Plasmopara viticola* (Downy mildew) and *B. cinerea* (Gray mold)	*Pseudomonas fluorescens* PTA-CT2 (Grapevine rhizosphere)	Systemic resistance. *P. viticola*: Stilbenes accumulation. Upregulations of *PR1*, *PR2*, *GST*, *ACO*, and *HSR*. *B. cinerea*: Stilbenes and resveratrol accumulation. Upregulations of *ACO*, *PR1*, *GST* genes and *HSR* downregulation.	Greenhouse, 2 years-old cv. Pinot noir-5BB and Solaris30-5BB (Soil drenching)	(Lakkis *et al*., 2019) [108]
*E. necator* (Powdery mildew)	*T. harzianum* 5R (Citrus rhizosphere, *Trichoderma viride* F-01812 (sugarcane soil), and F-01951 (forest soil), and *T. asperellum* F-01769 (soil)	Systemic resistance. Increase in total phenol contents, chitinase, and β-1,3-glucanase in leaves.	Field, 8 years-old cv. Centennial Seedless (Soil drenching)	(Sawant *et al*., 2020) [109]
*Phaeomoniella chlamydospora* (Esca)	*Pythium oligandrum* Oth-2, Oth-3, Sto-1, Oth-4, Sto-7, and Sto-11 (Grapevine rhizosphere)	Systemic resistance. Oligandrin synthesis *in vitro*. *PR10*, *Glu*, *Gst*, and *Lox* upregulations.	Greenhouse, 4 months-old cv. Cabernet Sauvignon (Collar inoculation)	(Yacoub *et al*., 2016) [110]
*Neofusicoccum parvum* (Botryosphaeria dieback)	*B. subtilis* PTA-271 (Grapevine rhizosphere), and *Trichoderma atroviride* SC1 (Hazelnut wood)	Decrease of salicylic acid (SA)-dependent defenses compared to symptomatic non plants. LOX9, PR2, PAL, and STS upregulation in leaves.	Culture chamber, 1 year-old cv. Chardonnay and Tempranillo. Soil drenching (*B. subtilis* PTA-271) and wound painting (*T. atroviride* SC1).	(Leal *et al*., 2021) [111]
*Agrobacterium tumefaciens* (Crown gall)	*Pseudomonas kilonensis* Sn48, (Grapevine roots) and *P. agglomerans* Sa14 (Wild-grapevine stem)	Systemic resistance. Stilbenic phytoalexins (*trans*-resveratrol, *trans*-piceid, and ε-viniferin) global accumulation in leaves, roots, and stems. *PR1*, *PR2*, and *PR4* genes upregulation in leaves.	Greenhouse, 4 weeks-old cv. Chardonnay (Root dipping)	(Asghari *et al*., 2020) [112]

### Microbial diversity as a biological marker for grapevine fitness

Many biotic and abiotic stresses occur in vineyards and can lead to plant decline or dieback if not managed properly. Grapevine dieback afflict viticulture worldwide and can be defined as a pluriannual decrease in vine productivity linked to its sudden premature or gradual death due to environmental causes and/or agronomic practices [72]. Despite evidence of negative impact on microbial communities in young replanted vines due to long-term monoculture and intense replanting management, replacing the dead vines with young vines remains sometimes the only solution to palliate this problematic dieback [73, 74]. Grapevines are a perennial plant which require significant time-consuming cultivation; at least three years are needed for the new plant to harbor productive grapes [75]. To this end, accelerating the growth of young cuttings with plant growth-promoting rhizobacteria (PGPR) or AMF may be an interesting approach to compensate for the lack of productivity during the beginning of replantation, however this approach has not been widely studied in vineyards [76]. However, this strategy may increase the incidence and severity of grapevine trunk diseases (GTDs) symptoms due to the predisposition of GTD to affect such vineyards managed using training and pruning techniques which promote vine growth [77]. On that account, microbiome engineering which is an actual trend which encompasses crops and numerous cultivars [78], appears to be a promising strategy against environmental stressors. Microbiome engineering often refers to a set of tools which strengthen the soil microbiome and hence the plant-associated microbiome through nutrient uptake and pathogen control (Fig 2). Among these tools, agricultural practices (*e.g.* cover crop, irrigation, tillage), soil amendment, and plant material choice (*i.e.* grafted rootstock or not) can interfere with microbial diversity which is considered as a key biomarker in plant protection and growth strategies [79]. The greatest microbial diversity was found in organic vineyards compared to conventional ones [80] but with a lower soil microbial biomass [81]. This difference in diversity may be related to the abundance of organic matter which are a rich source of exogenous microbial inoculants which can colonize the vines. A meta-analysis made by Karimi *et al.* (2020) [82] highlighted the effect of viticultural practices on soil microbiological diversity and showed that tillage, absence of cover crop, and mineral fertilization all contributed significantly to reductions in soil biodiversity. Microbiome inoculation is another interesting tool that directly modify the soil and/or rootstock microbiome functionalities and compositions.

**Table 4:** List of inocula used for their beneficial effect on grapevine submitted to abiotic stress and applied on the soil or root system

**Abiotic stress** **(Factor to counter)**	**Inoculum identification (Origin)**	**Observations**	**Plant material** **(Type of application)**	**Reference**
Arsenic	*Bacillus licheniformis* Rt4M10, *Micrococcus luteus* Rz2M10 and *P. fluorescens* Rt6M10 (Grapevine root endosphere and rhizosphere)	Reduction of arsenic toxicity indicators with enhanced ascorbate peroxidase activity (*B. licheniformis*) and increased peroxidase activity (*Micrococcus luteus* and *P. fluorescens*)	Greenhouse, 2 years-old cv. Malbec (Leaf sprayed and stem-based inoculation)	(Funes Pinter *et al*., 2018) [117]
Drought	*Acinetobacter* and 2 *Pseudomonas* spp. (Grapevine root endosphere)	Higher tolerance to water deficit by maintaining photosynthetic activity and growth which was rootstock dependent. Positive effect on evapotranspiration and stomatal conductance.	Greenhouse, 1 year-old cv. SO4, 420A, 5BB (Roots dipping) Field, 1 year-old cv. Barbera (Roots dipping)	(Rolli *et al*., 2015) [118]
Drought	*Glomus mosseae* (not specified)	Higher tolerance to water deficit by maintaining photosynthetic activity and growth which was rootstock dependent. Positive effect on evapotranspiration and stomatal conductance. Increase of phosphorus content in leaves.	Greenhouse, 1 year-old cv. Cabernet-Sauvignon grafted on 110R, 41B, 1103P, 5BB, 44–53 Malegue, 140R and 101–14MGt (Soil inoculation)	(Nikolaou *et al*., 2003) [119]

### Biological control agents (BCAs) as limited but efficient disease management strategies

Nurseries have proposed to winegrowers the possibility of inoculating rootstocks with specific microorganisms such as AMF prior to planting, in an effort to improve grapevine resilience to abiotic and biotic stresses. Biological control provides tools for disease management which are partly based on soil microbial properties that promote plant health and fruit quality. This strategy called biocontrol, has been exploited recently as an alternative to synthetic or chemical pesticides [83]. The most common BCAs in viticulture are used in spray application and are partly efficient, compared to the synthetic solutions, against powdery, downy mildew or gray mold, caused by *Erysiphe necator*, *Plasmopara viticola*, and *Botrytis cinerea* respectively [84]. Currently, commercial microbial fungicides sprayed on the grapevine aerial part can be derived from bacteria, yeast, and multicellular fungi (Table 2). Those listed microorganisms are present in a variety of habitats worldwide, and can naturally be found in vineyard soils [85–88], hence comforting the vineyard soil studies for BCA screening.

Usually, spray applications are applied on the aerial part of the vine, targeting the leaves and berries where the first symptoms of the disease occur. However, the vine architecture and dense foliage may reduce the efficiency of the product, allowing the pathogen to sporulate on the untreated part of the crop. One solution to counteract the pathogen growth in viticulture is to leverage the microbe-associated molecular patterns (MAMPs) from beneficial microbes through belowground host-specific receptors, which prime grapevine immune response [97]. This strategy is referred to as induced systemic resistance (ISR) and can benefit both the aboveground parts of the plant and the roots via BCAs when applied to the soil or grapevine root system (Table 3). ISR leads to the production of phytoalexins and/or pathogenesis-related (PR) proteins in the distancial parts. Phytoalexins are low weight metabolites synthesized after microbial recognition and signaling in plant cells acting as defense compounds. In grapevines, these molecules (Table 3) are mainly stilbenes and encompasses *trans*-resveratrol, *trans*-ε-viniferin, and its derivative *trans*-piceid [98]. Moreover, it has been shown that the BCA oomycete *Pythium oligandrum* inoculated at the root level can modulate the transcriptome of the grapevine but also of the *Phaeomoniella chlamydospora* virulence factors, a GTD ascomycota fungus, even when the two microorganisms are not in direct contact [99]. Among the GTDs, black-foot and Petri diseases are the most common and are present in nurseries and young vineyards. Their symptoms in fields include overall reduced growth, dysregulation in the budbreak and sprouting, with chlorotic leaves and necrosis on the rootstock [100]. *Trichoderma* spp., *Bacillus*, and *Pseudomonas*-based commercialized products as well as two potential BCAs (*i.e. P*. *oligandrum* Po 37, *Streptomyces* sp. E1 and R4) reduced the Black-foot and Petri diseases by dipping the roots before planting under field conditions [101]. Stempien *et al.* (2020) unveiled the grapevine defense activation triggered by *Trichoderma atroviride* (T-77 and USPP T1) drenching and its colonization on rootstock cultivars 110R, US 8–7, 1103P. It appeared that the level of expression of genes such as *VvSTS* and *VvChit4c* encoding proteins involved in stilbene synthesis and chitinase, respectively, was dependent on the rootstock genotype and *Trichoderma* strain used. Recently Jaarsveld *et al*. (2021) [102] showed the higher colonization capacity by six *Trichoderma* products on graftlings (Sauvignon blanc cv. Grafted onto Ramsey) basal ends compared to middle or root tip part, even though *Trichoderma* spp. treatments were not sufficient to prevent fungal infections. Clear evidence of the biocontrol effects was observed *in vitro*, in greenhouse and in field (Table 3). These findings suggest that preventive application by soil drenching or root inoculation could be a promising strategy for disease management since the molecular mechanisms underlying the biocontrol effects of the inoculum are deciphered.

### Microbiome can enhance abiotic stress tolerance

By mitigating abiotic stresses, microbiome × rootstock interactions could be a relevant way to contribute to adaptation in the global climate change context. Up to now, the mechanisms developed by the plants to recruit their microbiomes in response to specific abiotic stresses remain poorly understood.

The root microbiome can enhance water deficit tolerance by acting in hormone regulation or by increasing plant antioxidant activity [113]. To this end, trends in microorganisms’ biomass, diversity, and activity under water deficit conditions have been explored [113, 114]. Exopolysaccharides (EPS) allow beneficial microbes to efficiently colonize the rhizosphere by increasing the percentage of stable soil aggregates and thus by increasing water and nutrient uptake [114]. It was also demonstrated that microorganisms from more fluctuating environments have a higher functional acclimatization [115]. In addition, plants benefit from their associated microbiome to tolerate water deficit, especially when the microbiome has been previously exposed to water deficit with the host plant in years before [116]. In grapevines, few studies have been made on the microbiome impact on abiotic stress [30]. However all the microorganisms tested were originated from root endosphere compartment and some of them vary in their effect depending on the rootstock genotype (Table 4). This comforts the hypothesis that microbiota from resistant rootstock in stressed environment might be an interesting strategy to investigate.

In addition, several microorganisms isolated from grapevine roots were studied for their capacity to synthetize protective molecules that might alleviate abiotic stresses. Carotenoids, known for their antioxidant activities and as precursors of abscisic acid (ABA), were produced by *Microbacterium imperial* Rz19M10, *Kocuria erythromyxa* Rt5M10, and *Terribacillus saccharophilus* Rt17M10 [120] but also by *Bacillus licheniformis* Rt4M10 [121]. The metabolism of ABA could be modulated in the advantage of inoculated grapevines with arbuscular mycorrhizal symbiosis [45]. Among the protective molecules, the melatonin allows to counteract the negative effects of abiotic stresses and it has been shown that inoculated grapevines with *Bacillus amyloliquefaciens* SB-9 [122] or with *Pseudomonas fluorescens* RG11 [123] accumulate more melatonin. Additionally to bacterial endophytes, water deficit stress can be alleviated by the presence of AMF thanks to their external mycelium that increase water use efficiency even though there is no current evidence of direct water transfer to the plant [54].

Besides the issues surrounding water deficit, the problem of soil salinization impacts a large percentage of irrigated vineyards worldwide [124]. AMF are known to improve growth related traits in saline conditions. Khalil (2013) [125] demonstrated on three rootstocks genotypes (1103P, Harmony, and Dogridge) that AMF addition contributes to increase plant height, stem diameter, leaf area, total leaf number, and total dry weight even if the effects were not significant. The total carbohydrates, leaf free proline content, and total leaf chlorophyll content were higher in inoculated seedlings than in uninoculated ones, suggesting a higher osmoprotection coupled to a photosynthesis maintenance. Moreover, mycorrhizal inoculation tends to decrease the Na and Cl concentrations while increasing P and K leaves content. A relevant choice of rootstock with mycorrhizal inoculation could be one way to avoid salinity problems in a vineyard.

The complexity of the interactions between the plant, the microbiome, and the surrounding environment is an issue that must be overcome to understand the beneficial associations between plants and microbes. It appears more relevant to isolate plant growth-promoting microbe (PGPM) that can promote tolerance to a specific abiotic stress from environments in which this stress occurs [126]. It could be outstanding to study the plasticity of the PGPM to rootstock × scion × interactions at the field level, hence the importance of including the microbiome in grapevine breeding programs [127]. As suggested for the tree species, association of rootstocks with different beneficial microbiota could be a relevant way to share the benefits of the microbiota from one individual to another to get a “microbial complementarity” [128].

### Are soil microbial inoculum a safe and relevant process to increase grapevine resilience?

The establishment and persistence of the BCAs in the soil and root compartments remain one of the most important concerns in microbial inoculant preparation [129]. Although the transfer of inoculation to different climatic regions can be a success, the effect may not be the same depending on pedoclimatic features [130]. Aside from these technical aspects, the BCAs legislation among EU, USA, and worldwide markets are quite different but remain important for their biosafety which are based on molecular identification coupled to pathogenicity, toxicological, and 37°C-growth tests [131]. While the biosafety issue has always been evaluated for human healthcare and plant health, the mass application of PGPM in the environment is never considered during the BCAs development. What if the PGPM application provokes soil or plant microbiome dysbiosis and lately its degradation [132]? What if a BCA turns out to become pathogenic, due to horizontal gene transfer from other surrounding microbes or because of the evolution or speciation?

In grapevine wood tissues, Haidar *et al.* (2021) [132] unveiled the synergistic effect of some bacterial strains with the basidiomycete *Fomitiporia mediterranea* involved in esca complex, to degrade wood components. The interesting part is the capacity of some of these bacterial strains to inhibit the pathogen growth *in vitro*, while having cellulose and xylan degradation properties. In grapevines, colonization process by inoculating beneficial endophytes such as *Paraburkholderia phytofirmans* strain PsJN or strains of *Enterobacter ludwigii* and *Pantoea vagans* have been studied in young plants [134, 135], and among the PGPR inoculated on grapevine roots, they are mainly composed from *Pseudomonas*, *Bacillus*, *Pantoea*, and *Burkholderia* genera (Table 3). However, depicting the PGPR inoculation impact on the soil microbiome remains a challenge and should combine both culture-dependent and independent approaches. Indeed, exogenous microorganisms might affect soil quality negatively by modifying soil capacity to process bio-geochemical cycles and hence, its potential to promote vine growth.

Soil exhibits the natural ability to suppress disease through its microbiome composition which is enhanced by agricultural processes that positively influence microbial diversity [136, 137]. For instance, Nerva *et al.* (2019) [138] investigated the microbial profile of both Esca-symptomatic and asymptomatic soils which suggested that higher proportions of *Curvularia*, *Coprinopsis*, *Bacillus*, and *Streptomyces* genera could suppress disease symptoms. These studies further support the idea that bulk soils are a major source of inoculum for pathogens. Microbial transplant is now assumed in medical research as a solution to modulate the human microbiota coupled to therapeutic effects [139]. While not conducted in a vineyard, Siegel-Hertz *et al.* (2018) [140] used soil transplants from suppressive soil to show inhibiting effects on Fusarium wilt conductive soils. Exclusive bacterial and fungal genera were found in Fusarium wilt-suppressive soils compared to conducive soils which suggest that microbiome transplant could be an efficient and promising way to promote microbiome diversity. This strategy within a vineyard could counteract the microbiome dysbiosis and the problematic effect of the inoculum survival since the soils possess quite similar abiotic features.

Biocontrol is assumed to be less efficient in disease management compared to chemical and synthetic products. One biotechnology-based tool that must be mentioned for increasing the microorganisms’ efficiency in pathogen control is the protoplast fusion technique, which is mainly studied for genetic transformation and somatic hybridization. This approach is quite difficult in grapevines and has recently been used for whole grapevine generation from protoplasts [141]. Protoplast fusion technique is also used in PGP and biocontrol bacteria to merge distinct traits. For instance, Gaziea *et al.* (2020) [142] attempted to merge, the biocontrol ability of *Bacillus thuringiensis* I977 against *Meloidogyne* spp. and the PGP capacity of *Pseudomonas aeruginosa* in grapevine seedlings and successfully controlled the root-knot nematode while promoting the plant growth. While this approach has not been tested on the field, it has already been considered against root-knot nematodes [143] and remains an interesting solution for BCA or biofertilizer products. *Trichoderma* spp., which are one of the most famous BCAs worldwide, have also been subjected to capacity enhancement for soil-borne disease suppressiveness [144]. Strains engineered via protoplast fusion are not affiliated to genetically modified organisms’ regulations since this technique is a form of natural homologous recombination [145], hence giving the possibility for BCAs to have more positive impacts on grapevine health.

## Conclusions and future prospects

Altogether, these findings demonstrate that the grapevine is able, via rootstock and scion genotypes, to select distinct but potentially beneficial microorganisms close to the roots. Although there is no consensus regarding the choice of hypervariable regions to amplify and sequence (Table 1), it is still possible to make comparable taxonomic descriptions between studies at the phyla level. However, it may be quite difficult to compare at the genera or species level since bias, in addition to “universal primers” choice, can occur until data processing [146]. The rhizosphere and root-associated microbiome, which are a balance between stress and fitness, would be relevant biological indicators of plant health status. The rhizosphere could be considered as an extended root phenotype, presented by Dawkins (1982) [147], which is a trait that may also reflect the agronomic properties of the rootstock as well as its health status. To this end, soil microbial diversity could explain many dysbiosis and symbiosis observed in the grapevine organs since most of them are recruited from the surrounding soil. Until now, no research of soil virome in vineyards has been done even though it is known that the viruses are playing important roles in ecological processes and microorganism evolution [148], whereas the grapevine associated virome has been well investigated in leaf and trunk tissues [149].

Given increasing environmental constraints, improving viticulture sustainability is currently a major challenge. One important area of study to improve sustainability includes better understanding soil microbiome functionalities and its effects on the grapevine metabolism and agronomic responses. Based on the current literature, the soil microbiome could offer new engineering solutions to palliate intensive phytosanitary use and climate change issues. To this end, molecular and microbial dialogues between the scion and the soil through the rootstock must be considered. The core microbiome of the grape should be preserved as it represents a sensitive balance for the plant protection, growth, nutrition, and health.

## Supplementary Material

Web_Material_uhac019
