# Supplementary material for: Grapevine rootstock and soil microbiome interactions: Keys for a resilient viticulture
Source: Hortic Res. 2022 Feb 19;9:uhac019. doi: 10.1093/hr/uhac019 (PMC8985100; doi:10.1093/hr/uhac019)
Supplement: Web_Material_uhac019 [file web_material_uhac019.docx]

**Table S1**: Examples of the bacterial diversity among the different grapevine compartments

| Compartments | Approaches used and main phyla detected | Main microorganisms detected | Scion/Rootstock combinations | References |
| --- | --- | --- | --- | --- |
| Berry surface, leaves | MiSeq on 16S rRNA (V3-V4 regions)  Leaves: *Proteobacteria*, *Bacteroidetes*, *Actinobacteria*  Berry surface: *Proteobacteria*, *Firmicutes*, *Actinobactaria* | Leaves: *Alphaproteobacteria*, *Saprospirae*, *Cytophagia*, *Actinobacteria*  Berry surface: *Alpha*/*Beta*/*Gamma*-*Protecobacteria*, *Bacilli*, *Actinobacteria* | Dolcetto, Sangiovese cv. grafted on different rootstocks (not specified) | (Vitulo *et al.*, 2019)^155^ |
| Berry surface, grape must, leaves, wine, soil | MiSeq on 16S rRNA (V4 region)  Berry surface and must, leaves, wine: *Proteobacteria*, *Firmicutes*  Soil: *Proteobacteria*, *Firmicutes*, *Bacteroidetes*, *Actinobacteria* | Berry surface: *Pseudomonas*, *Acinetobacter*, *Kaistobacter*, *Sphingomonas*  Grape must: *Oenococcus*, *Pseudomonas*  Leaves: *Pseudomonas*, *Acinetobacter*, *Kaistobacter*, *Sphingomonas*, *Oenococcus*  Wine: *Oenococcus*  Soil: *Kaistobacter*, *Arthrobacter*, *Skermanella*, *Sphingomonas* | Cabernet Sauvignon, not specified if grafted or not | (Wei *et al.*, 2018)^13^ |
| Soil, roots, graft union, cane | MiSeq on 16S rRNA (V4 region)  Soil: *Proteobacteria*, *Acidobacteria*, *Actinobacteria*, *Bacteroidetes*, *Firmicutes*, *Crenarchaeota*, *Planctomycetes*, *Verrucomicrobia*, *Chloroflexi*  Root: *Proteobacteria*, *Actinobacteria*, *Bacteroidetes*  Graft union: *Proteobacteria*, *Actinobacteria*  Cane: *Proteobacteria* | Soil: *Nitrososphaera*, *Flavobacterium*, *Agrobacterium*  Roots: *Methylobacterium*, *Pseudomonas*, *Steroidobacter*, *Erwinia*, *Sediminibacterium*, *Bradyrhizobium*  Graft union: *Pseudomonas*, *Agrobacterium*, *Erwinia*, *Sodalis*  Cane: *Pseudomonas* | Cabernet Dorsa grafted onto SO4 | (Faist *et al.*, 2016)^156^ |
| Berry surface | Ion Torrent on the 16S rRNA (V4 region)  *Firmicutes*, *Proteobacteria* | *Bacillus*, *Erwina*, *Acinetobacter*, *Oenococcus* | Ungrafted Carignan and Grenache cultivars | (Portillo *et al.*, 2016)^157^ |
| Bulk soil, rhizosphere, roots, leaves, grape surface, must, flowers | MiSeq on 16S rRNA (V4 region)  Soil: *Proteobacteria*, *Acidobacteria*, *Bacteroidetes*, *Verrucomicrobia*, *Planctomycetes*  Roots: *Proteobacteria*, *Acidobacteria*, *Bacteroidetes*, *Verrucomicrobia*, *Actinobacteria*  Leaves, grapes, flowers: *Proteobacteria* | Roots: *Xanthomonadales* (*Steroidobacter*), *Cytophagaceae*, *Chitinophagaceae*, *Rhizobiales*, *Actinomycetales*  Leaves, grape: *Sphingomonas*, *Pseudomonas*, *Methylobacterium*  Flowers: *Pseudomonas* and *Erwinia* spp. | Merlot cv. grafted onto 3309C | (Zarraonaindia *et al.*, 2015)^18^ |
| Grape must, wine | MiSeq on 16S rRNA (V4 region)  Grape must: *Firmicutes*, *Proteobacteria*  Wine: *Firmicutes* | Grape must: *Leuconostocaceae*, *Enterobacteriaceae*, *Bacillaceae*, *Pseudomonas*, *Sphingomonas*  Wine: *Leuconostacaceae*  Grape must: *Botryotinia fuckeliana*, *Cladosporium*, *S. cerevisiae*  Wine: *Cladosporium*, *Botryotinia fuckeliana*, *S. cerevisiae* | Cabernet Sauvignon, Chardonnay, Zinfandel cv., not specified if grafted or not | (Bokulich *et al.*, 2014)^158^ |
| Leaves | Pyrrosequencing 16S rRNA (V5-V9 regions)  *Proteobacteria*, *Actinobacteria* | *Pseudomonas*, *Frigoribacterium*, *Sphingomonas*, *Erwinia*, *Acetobacter*, *Curtobacterium* | Pinot gris cv., not specified if grafted or not | (Perazzolli *et al.*, 2014)^159^ |
|  | Pyrrosequencing 16S rRNA (V6 region)  *Proteobacteria*, *Actinobacteria* | *Enterobacteriaceae*, *Pseudomonadaceae*, *Moraxellaceae*, *Comamonadaceae*, *Streptococcaceae*, *Actinobacteria* | Tempranillo cv., not specified if grafted or not | (Pinto *et al.*, 2014)^160^ |

**Table S2**: Examples of the fungal diversity among the different grapevine compartments

| Compartments | Approaches used and main phyla detected | Main microorganisms detected | Scion/Rootstock combinations | References |
| --- | --- | --- | --- | --- |
| Rootstock xylem | MiSeq on ITS2  *Ascomycota* | *Cladosporiaceae*, *Dothioraceae*, *Nectriaceae*, *Pleosporaceae*, *Ploettnerulaceae*, *Trichocomaceae* | Ungrafted 110R and 41B | (Gramaje *et al*., 2021)^161^ |
| Roots, rhizosphere | MiSeq on ITS2,  *Ascomycota*, *Basidiomycota* | *Nectriaceae*, *Ceratobasidiaceae*, *Mortierellaceae* | Ungrafted SO4 | (Carbone *et al*., 2021)^71^ |
| Grapes, flowers, leaves, roots | MiSeq on ITS1  *Ascomycota*, *Basidiomycota* | *Aureobasidium, Cladosporium, Epicoccum, Mortierella, Cryptococcus, Debaryomyces, Saccharomyces, Mycosphaerella, Lophiostoma, Alternaria, and Penicillium* | Pinot Noir cv., not specified if grafted or not | (Liu & Howell, 2021)^20^ |
| Roots, bulk soil, rhizosphere | MiSeq on ITS2  *Ascomycota*, *Basidiomycota* | *Mortierellaceae*, *Nectriaceae*, *Pleosporaceae* | Tempranillo cv. grafted onto 110R | (Martínez-Diz *et al*., 2019)^25^ |
| Branches | Plating method  *Ascomycota*, *Basidiomycota* | *Alternaria* spp., *Aureobasidium pullulans*, *Diplodia seriata*, *Cladosporium* spp., *Epicoccum nigrum* | Riesling cv., not specified if grafted or not | (Kraus *et al.*, 2019)^162^ |
| Berry surface | Miseq on ITS  *Ascomycota*, *Basidiomycota* | *Pleasporaceae*, *Cladosporiaceae*, *Sporidiobolales*, *Aureobasidiaceae* | Table grape Crimson Seedless cv., not specified if grafted or not | (Carmichael *et al.*, 2019)^163^ |
| Total berry, flower, leaves, air | Plating method and MiSeq on ITS1 / ITS4  *Ascomycota*, *Basidiomycota* | Plating: *Alternaria*, *Cladosporium*, *Aspergillus*, *Botryosphaeria*  MiSeq: *Alternaria*, *Cladosporium*, *Pleosporaceae*, *Lasiodiplodia*, *Phoma*, *Botrytis*, *Aspergillus*, *Penicillium* | Midnight beauty cv. | (Dissanayake *et al.*, 2018)^164^ |
| Berry surface, grape must, leaves, soil | MiSeq on ITS  All compartments: *Ascomycota*, *Basidiomycota* | Berry surface: *Aureobasidium*, *Pleosporaceae*, *Dothideale*, *Cryptococcus*  Grape must: *Aureobasidium*, *Erysiphe*, *Aspergillus*, *Cryptococcus*  Leaves: *Aureobasidium*, *Pleosporaceae*, *Dothideales*, *Erysiphe*, *Alternaria*, *Cryptococcus*  Soil: *Ascomycota*, *Sordariales*, *Tetracladium*, *Dothideales*, *Pleosporales* | Cabernet Sauvignon cv., not specified if grafted or not | (Wei *et al.*, 2018)^13^ |
| Wood: crown area and grafting area from rootstock | Plating Method and MiSeq on ITS2 from cDNA | MiSeq: *Erysiphaceae*, *Lasiosphaeriaceae*, *Mycosphaerellaceae*, *Nectriaceae*, *Pleosporaceae*  Plating: *Bionectriaceae*, *Nectriaceae*, *Microascaceae*, *Trichocomaceae* | Garnacha Tintorera cv. grafted onto rootstock 110R, and Sauvignon Blanc cv. grafted onto SO4 | (Eichmeier *et al*., 2018)^165^ |
| Grape must | MiSeq on ITS  *Ascomycota*, *Saccharomycotina* | *Cladosporium* spp., *Botrytis cinerea*, *Penicillium* spp., *Davidiella tassiana*, *Aureobasidium pullulans*, *S*. *cerevisiae*, *Hanseniaspora uvarum*, *Candida zemplinina* | Cabernet Sauvignon, Chardonnay, Zinfandel cv., not specified if grafted or not | (Bokulich *et al.*, 2014)^158^ |
| Leaves | Pyrrosequencing on ITS *Ascomycota*, *Basidiomycota* | *Cryptococcus*, *Dioszegia*, *Sebacina* | Pinot gris cv., not specified if grafted or not | (Perazzolli *et al.*, 2014)^159^ |
|  | Pyrrosequencing on ITS2 and D2  *Ascomycota*, *Basidiomycota* | *Rhizopus*, *Mucor*, *Zoophthora*, *Pandora*, *Aureobasidium*, *Sporormiella*, *Alternaria* | Tempranillo cv., not specified if grafted or not | (Pinto *et al.*, 2014)^160^ |
